# Performing a shortened version of the Action Research Arm Test in immersive virtual reality to assess post-stroke upper limb activity

**DOI:** 10.1186/s12984-022-01114-3

**Published:** 2022-12-03

**Authors:** Quentin Burton, Thierry Lejeune, Stéphanie Dehem, Noémie Lebrun, Khawla Ajana, Martin Gareth Edwards, Gauthier Everard

**Affiliations:** 1 Neuro Musculo Skeletal Lab (NMSK), Institut de Recherche Expérimentale et Clinique, Secteur des Sciences de la Santé, Université catholique de Louvain, Brussels, Belgium; 2 Psychological Sciences Research Institute (IPSY), Université catholique de Louvain, Louvain-la-Neuve, Belgium; 3 Service de médecine physique et réadaptation, Cliniques universitaires Saint-Luc, Brussels, Belgium; 4 Louvain Bionics, Université catholique de Louvain, Louvain-la-Neuve, Belgium; 5 Cliniques universitaires Saint Luc, Médecine Physique et Réadaptation, Avenue Hippocrate 10, 1200 Brussels, Belgium

**Keywords:** Stroke, Virtual reality, Upper extremity, Remote sensing technology, Patient outcome assessment

## Abstract

**Background:**

To plan treatment and measure post-stroke recovery, frequent and time-bounded functional assessments are recommended. With increasing needs for neurorehabilitation advances, new technology based methods, such as virtual reality (VR) have emerged. Here, we developed an immersive VR version of the Action Research Arm Test (ARAT-VR) to complement neurorehabilitation.

**Objective:**

This study aimed to assess the validity, usability and test–retest reliability of the ARAT-VR among individuals with stroke, healthcare professionals and healthy control subjects (HCS).

**Methods:**

Among the 19 items of the ARAT, 13 items were selected and developed in immersive VR. 11 healthcare professionals, 30 individuals with stroke, and 25 HCS were recruited. Content validity was assessed by asking healthcare professionals to rate the difficulty of performing each item of the ARAT-VR in comparison to the classical Action Research Arm Test (ARAT-19). Concurrent validity was first measured using correlation (Spearman tests) between the ARAT-VR and ARAT-19 scores for the individuals with stroke, and second through correlation and comparison between the scores of the ARAT-VR and the reduced version of the ARAT (ARAT-13) for both individuals with stroke and HCS (Wilcoxon signed rank tests and Bland–Altman plots). Usability was measured using the System Usability Scale. A part of individuals with stroke and HCS were re-tested following a convenient delay to measure test–retest reliability (Intra-class correlation and Wilcoxon tests).

**Results:**

Regarding the content validity, median difficulty of the 13 ARAT-VR items (0[0 to − 1] to 0[0–1]) evaluated by healthcare professionals was rated as equivalent to the classical ARAT for all tasks except those involving the marbles. For these, the difficulty was rated as superior to the real tasks (1[0–1] when pinching with the thumb-index and thumb-middle fingers, and 1[0–2] when pinching with thumb-ring finger). Regarding the concurrent validity, for paretic hand scores, there were strong correlations between the ARAT-VR and ARAT-13 (r = 0.84), and between the ARAT-VR and ARAT-19 (r = 0.83). Usability (SUS = 82.5[75–90]) and test–retest reliability (ICC = 0.99; p < 0.001) were excellent.

**Conclusion:**

The ARAT-VR is a valid, usable and reliable tool that can be used to assess upper limb activity among individuals with stroke, providing potential to increase assessment frequency, remote evaluation, and improve neurorehabilitation.

*Trial registration*https://clinicaltrials.gov/ct2/show/NCT04694833; Unique identifier: NCT04694833, Date of registration: 11/24/2020.

**Supplementary Information:**

The online version contains supplementary material available at 10.1186/s12984-022-01114-3.

## Background

Upper limb function is commonly affected after a stroke. Around 80% of individuals with stroke present with motor impairments and activity limitations of the upper limb [1]. To evaluate the severity of these impairments, and better predict individual recovery, experts recommend performing regular assessments of function, activity and participation (according to the International Classification of Functioning, Disability and Health model) [2, 3]. In clinical routine and research, the gold-standard assessment of post-stroke upper limb activity is the Action Research Arm Test (ARAT) [4–6]. This test has excellent clinimetric properties, consisting of manipulating objects of different sizes and shapes according to standardized instructions [7–12].

During the last decade, there has been rapid developments of technology that provide interesting new methods to deliver effective stroke rehabilitation [13]. Among these, virtual reality (VR) is one of the most used developments. VR applications may be categorized into immersive and non-immersive according to the input and output devices [14, 15]. Immersive VR refers to systems that fully immerse users senses into a virtual environment [16] through use of a head mounted display or, more rarely, using projections on a large and curved display with panoramic view [14, 16]. In immersive VR, participants mostly interact with the virtual environment using input devices such as controllers, joysticks or motion capture cameras [14]. In contrast, non-immersive VR refers to systems that generate a bidimensional virtual environment where users remain aware of the physical world [17] and participants interact with the virtual environment using a robotic device, controllers, a computer mouse, a trackpad, a tablet, etc. [14]. Non-immersive systems are generally displayed on output devices such as laptop, TV, or console screens [18, 19]. Both immersive and non-immersive VR systems are frequently associated with serious games to provide a realistic world experience through feedback and multisensorial stimulations, adapting exercise characteristics to the individual’s abilities, offering diverse possibilities to provide more entertaining therapy, enhance individual motivation [20], and to deliver home or self-rehabilitation [21, 22]. In terms of effectiveness, several meta-analyses have demonstrated that stroke rehabilitation interventions based on VR and serious game training induces a significant improvement of upper limb motor function and activity [23–26].

VR also has the potential to be used as an evaluation tool. As a human–machine interface, the VR system continuously collects a full range of interesting data such as interactive movement kinematics during its use. This offers the possibility to develop quantitative and objective measures that could be used autonomously and operated without the need of clinician presence. To date, several tests have been developed to measure post-stroke upper limb functions such as motor control [27] and manual dexterity in a virtual environment [28, 29]. For instance, in 2019, Kim et al. developed a non-immersive VR version of the Fugl-Meyer Assessment (FMA-UL) to assess post-stroke upper limb motor function [27]. Using a depth-sensing camera, they developed 13 of the 33 items of the FMA-UL and showed strong correlations between the FMA-UL VR version and the classical FMA-UL assessment. Regarding assessments of activity limitation, two studies developed the Box and Block Test in immersive VR [28–30]. The first study developed the test using controllers and data showed strong correlations between virtual and classical test scores when assessed among individuals with stroke [29]. The second study used hand-tracking technology and data showed moderate to strong correlations between scores when tested among a population of individuals with Parkinson disease [28]. The hand-tracking method enabled recording and the identification of participant hands and fingers using camera and infrared light emitting diodes. These optical sensors converted the images into electronical signals allowing to generate a virtual model of hand and finger movements using built in software. While such technology limits the provisioning of tactile feedback, it offers a more realistic and natural representation of hand and finger movements with objects. Hand-tracking technology therefore provides potential for improving assessments of fine manual dexterity and upper limb activity. However, to date, despite the potential of this technology, the ARAT has never been adapted and tested in immersive VR. In this context, we developed an immersive VR version of the Action Research Arm Test (ARAT-VR).

This study aimed to develop and validate an immersive virtual version of the ARAT among individuals with stroke and healthy control subjects (HCS). The hypothesis was that the ARAT-VR and ARAT scores would be correlated among individuals with stroke when performed with both the paretic and less affected hand. We also expected that HCS and individuals with stroke, would obtain similar scores on both tests when performed with the less-affected hand. Secondary objectives were to assess the usability and reliability of the ARAT-VR.

## Methods

### Study design and participants

This observational multicentric study involved individuals with stroke, HCS and healthcare professionals. All these participants were recruited in Cliniques universitaires Saint-Luc and Cliniques universitaires UCL Mont-Godinne (Belgium) between October 2021 and April 2022. The protocol was approved by their Ethics Committee and registered on clinicaltrial.gov (NCT04694833). Written informed consent was obtained from all participants after receiving information regarding the trial. This study followed the STROBE recommendations (Additional file 1).

Individuals with stroke were included if they were diagnosed as having a hemiparesis of the upper limb as a result of a stroke and had a corrected-to-normal vision. Upper limb hemiparesis was assessed with the self-adapting and Rash validated version of the FMA-UL [31]. Individuals with any other neurological or orthopedic pathology potentially affecting upper limb activity were excluded. Individuals with severe communication or cognitive impairments preventing the comprehension of simple instructions were also excluded. Individuals with stroke were classified according to delay between stroke onset and the day of the experiment: acute (< 15 days), subacute (15 days–6 months) and chronic stroke (≥ 6 months) [32].

HCS were recruited with normal or corrected-to-normal vision and were excluded if they presented with any neurological or orthopedic issues potentially altering their upper limb activity.

To assess the content validity of the ARAT-VR, we also recruited rehabilitation professionals. They were included if they had more than 3 years of experience in neurorehabilitation.

#### Materials

The ARAT consists of 19 items subdivided into four subtests: grasp, grip, pinch and gross movement [6]. For the grasp subtest, participants were asked to reach, grasp and lift wooden cubes (of various sizes and weight), a wooden sphere and a sharpening stone. For the grip subtest, participants had to pour water from one glass to another, to place tubes of various diameters and a ring onto aluminum pins. For the pinch subtest, participants were asked to grasp and displace marbles of different diameters with different fine pinch grips. Lastly, for the gross movement subtest, participants had to touch their neck, head and mouth with their contralesional hand (see Table 1, left and middle columns). All these subtests were performed according to standardized instructions and scored following an ordinal scale based on the quality and conclusion of the task execution: 3 points for correct and complete execution within the time limit (≤ 2.5 to 5 s depending on the task), 2 points for a complete execution requiring an unusually long time (> 2.5 to 5 s depending on the task), 1 point for a partial execution of the movement and 0 points when the movement was not initiated. The total score ranged from 0 to 57, where higher scores indicated better upper limb activity. In clinical routine, the ARAT duration is often reduced through the use of a decision tree that enables to skip intermediate items of each subtest when individuals obtain a maximal score on the most difficult item, or when individuals achieve a minimal score for the two easiest items of the subtest (Guttman scaling) [12]. In this study, participants were asked to perform all ARAT items to compare with those of the ARAT-VR. The total score was retrospectively computed using the same (Guttman scaling) method.

**Table 1:**
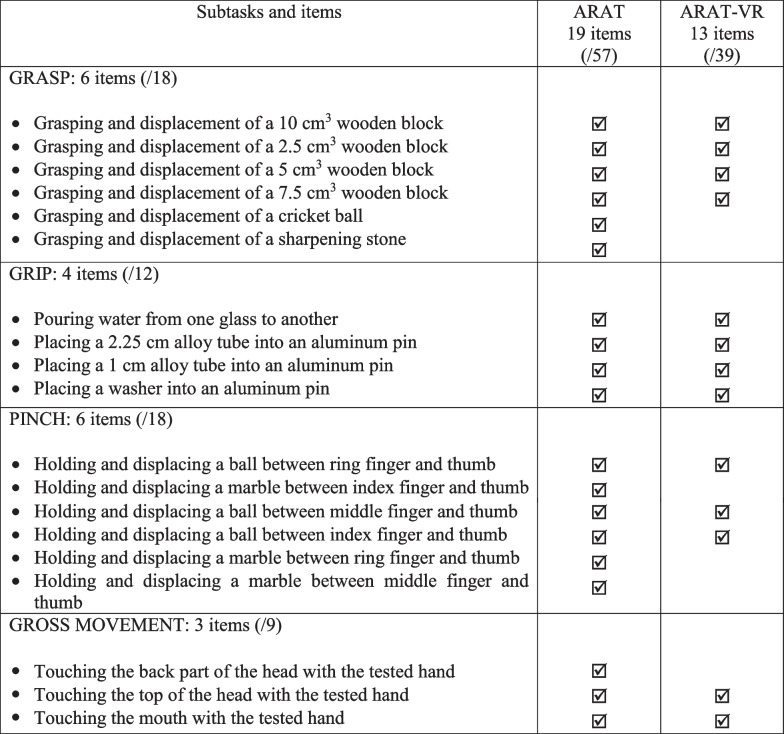
Items and subscales of the ARAT and ARAT-VR

The ARAT-VR was developed in C# language using Unity software 2020.1. The VR equipment consisted of a standalone virtual headset device (Oculus Quest 2®, Facebook) with 4 integrated infrared cameras capable to automatically detect the position and orientation of the individual’s hands and fingers. The headset was connected to a computer in order to stream the application, allowing the experimenter to see what the user was doing while performing the test. The ARAT-VR task contained 13 out of the 19 items of the ARAT, also subdivided in four subtests: grasp, grip, pinch and gross movement. Indeed, it was not possible to develop 6 ARAT items in VR because of limitations with the hand-tracking technology (see Table 1, right column). The current technology did not allow for accurate measures of grabbing a ball or a whetstone, and the individual was not able to perceive weight differences between objects. We therefore decided that the grasp item would only contain cubes. In addition, we only used the marbles of 1.5 cm diameter as the hand-tracking was not accurate enough to detect grip responses for the smallest marbles of 0.6 cm. Lastly, in the global movement subscale, the ARAT subtask of the individual placing their hand behind their head was removed because the helmet did not have cameras on the back. Moreover, in the ARAT-VR version, a longer time was allowed (10 s) for each item to obtain the maximal score (3). In a pretest, we observed that healthy participants required longer time to perform the items of the ARAT-VR compared to the ARAT. All items were rated: 3 (task completed in less than 10 s), 2 (completed between 10 and 30 s), 1 (initiated but incomplete movement between 30 and 60 s) and 0 (no movement possible or > 60 s). The total score ranged from 0 to 39, where a higher score indicated better upper limb activity.

As the ARAT-VR contained 13 items, the traditional ARAT score was computed in two different ways. The first consisted of computing the total score of the ARAT using a retrospective Guttman scaling (ARAT-19) and the second consisted of only scoring the 13 ARAT items that corresponded to the ARAT-VR (ARAT-13).

#### Procedure

**Fig. 1:**
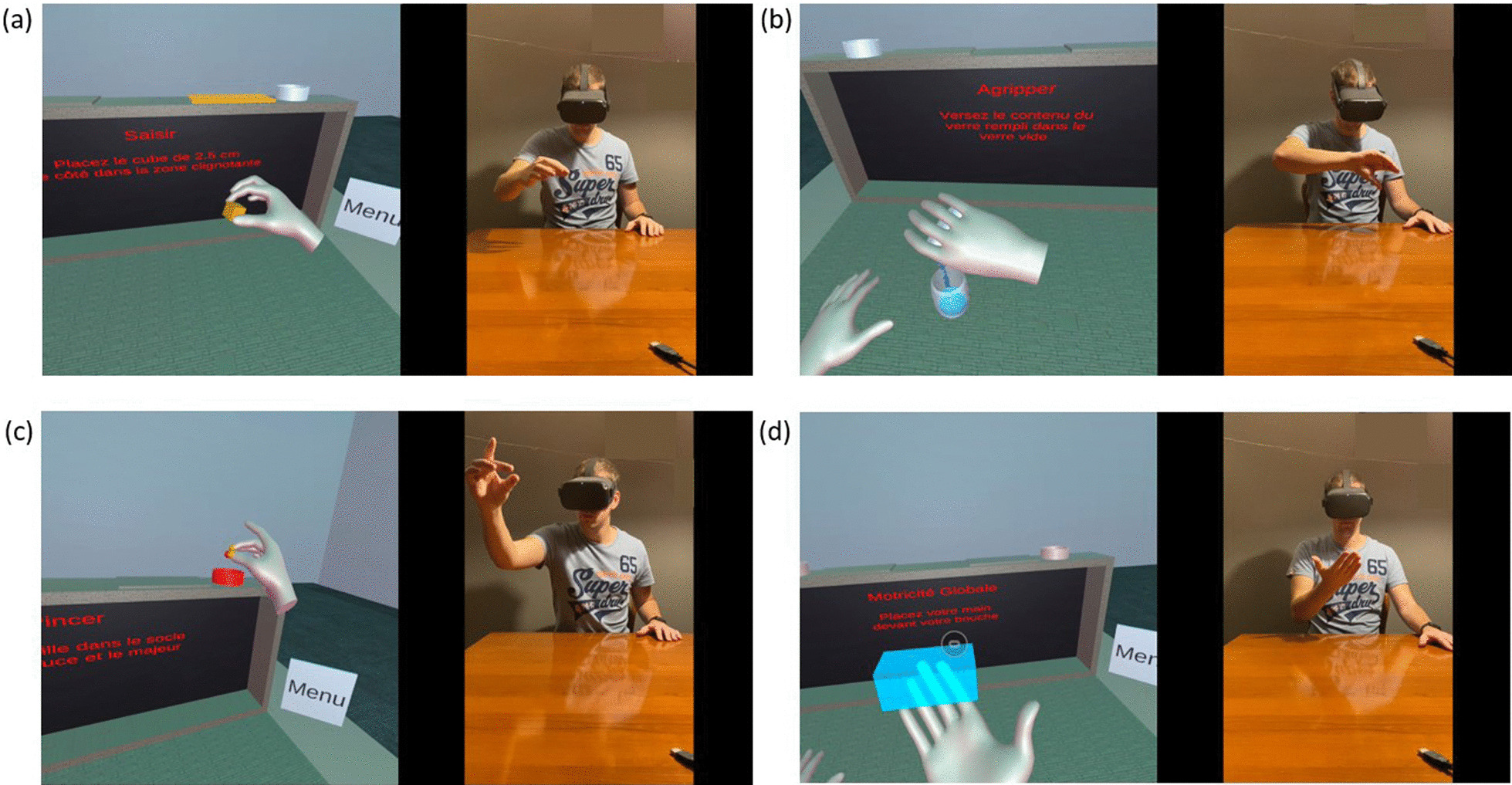
Representation of the ARAT-VR. This four-panel figure simultaneously represents the ARAT-VR environment displayed in the headset and the movements performed by a healthy control subject to realize the task when interacting with the system. **a** This panel represent the second item of the ARAT-VR and consists of grasping and displacing a virtual cube of 2.5 cm side. **b** This panel represents the fifth item of the ARAT-VR and consists of pouring water from one virtual glass to another. **c** This panel represents the eleventh item of the ARAT-VR and consists of grasping, holding and displacing a virtual marble with a thumb and middle finger pinching movement. **d** This panel represents the thirteenth and least item of the ARAT-VR and consists of touching the mouth (virtually represented by a blue rectangle target) with the tested hand

For both the ARAT and ARAT-VR, individuals were asked to sit on a chair, with their feet on the ground, and with a back support, without armrests. When performing the ARAT, the experimenter first explained the test to the participant to provide instructions for each task. When performing the ARAT-VR, the experimenter first set the basic settings and installed the headset. All individuals then benefited from a practice learning period corresponding to the achievement of all ARAT-VR tasks. After that, participants performed the test autonomously, following written or verbal instructions provided by the software application. Before and during each virtual task, written and verbal instructions were delivered to the individual by the software application through the headset. The procedure of the test and the dimensions of the virtual objects were similar to those of the classical ARAT. The height and position of the virtual table was adjustable to allow for matching with a real table. When the participant was ready to start a task, he or she was asked to put both hands on the table. A timer was then started. The grasping movement was initiated when the individual reached the object with a hand opening movement, where the distance between the thumb and fingers corresponded to the size of the object. Once grabbed, the object was released if the individual opened the aperture of their thumb and fingers, or brought the thumb and fingers closer together. The timer stopped when the task was successfully completed, or the time had elapsed, or the user chose to move to the next task (see Fig. 1 and a movie file, presented in Additional file 2, for more details). At the end of the test, an export file comprising the score and timing execution of each item was created by the software application and stored in the headset local memory.

After performing both the ARAT and ARAT-VR, healthcare professionals were asked to respond to two parts of a questionnaire (described in Additional file 3) to assess the content validity. In the first part, the professionals were asked to rate the difficulty of performing each item of the ARAT-VR in comparison to the classical ARAT. A Likert scale was used ranging from − 2 to 2 (with − 2 indicating that the sub-movement was much easier when performing the ARAT-VR than the classical ARAT; 0 indicating equivalence between the two versions, and + 2 indicating that the ARAT-VR was much more difficult than the classical ARAT). For each item, a median score with an interquartile range was computed from the sub-movement scores provided by all healthcare professionals. The second part of the questionnaire aimed to assess the ergonomic quality and the clearness of the explanations of the ARAT-VR only. Healthcare professionals were asked to give a general appreciation on a scale ranging from 1 to 5, where 1 point indicated no ergonomic quality and 5 points indicated strong ergonomic quality. A median score was then computed.

Concurrent validity was also evaluated to indicate the amount of agreement between the ARAT-19, ARAT-13 and ARAT-VR scores. All individuals and HCS were asked to respond to both the ARAT and ARAT-VR with both hands. The order of items in each test and the hand used by participants to start was randomized.

To assess the ARAT-VR usability, participants were asked to self-complete the System Usability Scale (SUS) [33]. This questionnaire comprises 10 items and aims to determine the subjective usability of the ARAT-VR.

To assess the test–retest reliability, a subgroup of the individuals with stroke and the HCS performed the ARAT-VR test a second time with the same assessor.

To assess the timing execution of the ARAT-VR, we scored the time made to perform each ARAT-VR item for each participant, as measured by the software application. A median score with an interquartile range was then calculated for the paretic hand.

#### Statistical analyses

Statistical analyses were performed using SIGMAPLOT 13.0 and R with alpha = 0.05. The sample size was determined to have a 0.5 correlation coefficient between the ARAT-19 and the ARAT-VR scores. The minimum number of individuals with stroke required was 30 to achieve 80% power with a 5% significance level.

Given that ARAT and ARAT-VR are ordinal scales, we only performed non-parametric tests. An ordinal logistic regression model was first conducted to identify all the factors potentially influencing the ARAT-VR score: ARAT, FMA-UL, age, sex, weight, height, type of stroke, side of stroke, time since stroke onset, the presence of cognitive impairments and the hand affected by the stroke (dominant vs. non-dominant). For individuals with stroke, Spearman coefficients were computed to evaluate correlations between the ARAT-VR and the ARAT-19 and ARAT-13 scores. A Bland–Altman plot was also performed to visualize the difference in ARAT-VR and ARAT-13 scores for the paretic hand. For both HCS and individuals with stroke, Wilcoxon-signed rank tests were conducted to compare the scores of the ARAT-VR and the ARAT-13 assessments. Lastly, to understand the influence of experience with technologies on VR performance, correlations between age and the difference between ARAT-19 and ARAT-VR scores were performed using Spearman correlation tests. Correlations were rated as small (0.1 < r ≤ 0.3), medium (0.3 < r < 0.5) or large (r ≥ 0.5) according to Cohen’s interpretation [34].

For the paretic hand, test–retest reliability was evaluated by performing a two-way mixed model Intraclass Correlation Coefficient (ICC) between the two repeated measures of the ARAT-VR. Reliability was rated as poor (ICC or r ≤ 0.40), moderate (0.40 < ICC or r < 0.75), or excellent (ICC or r ≥ 0.75)  [35]. Minimal detectable change (MDC) was computed for the ARAT-VR using the following calculation: 1.96 × standard error of measurement × √2. This provides the minimal magnitude of change to indicate true improvement, controlling for variability and measurement error [36, 37]. To assess equality between the first and second trial of the less-affected, dominant and non-dominant hand, Wilcoxon signed-rank tests were conducted.

## Results

Eleven healthcare professionals with a mean age of 30 ± 7.3 years took part in the trial. Among these, five were physical therapists, four occupational therapists and two doctors with specialization in physical medicine and rehabilitation. All were familiar with the ARAT before the experiment.

Twenty-five HCS with a mean age of 43 ± 20.0 years, and 30 individuals with stroke (22 males/8 females) with a mean age of 60 ± 10.9 years participated in the study. Individuals with stroke were recruited during acute (n = 8), subacute (n = 8) and chronic phase (n = 14) with a median time since stroke onset of 2.9[0.4–14.1] months. Complementary information is presented in Table 2.

**Table 2:** Participants’ demographic information, upper limb motor function and activity

	Patients with stroke (n = 30)	Healthy control subjects (n = 25)
Age (years)	59.8 ± 10.87	46.2 ± 23.23
Gender (M/F)	22/8	10/15
Dominant hand (L/R)	3/27	2/23
Time since stroke onset (months)	2.9[0.4–14.1]	/
Type of stroke (ischemic/hemorrhagic)	24/6	/
Side of stroke (L/R)	15/15	/
ARAT (/57)		
Paretic hand	48.5[20.5–54]	/
Less-affected hand	57[56.75–57]	/
Fugl-Meyer (%)	82.5[75–90]	/

*M* male, *F* female, *R* right, *L* left

### Primary outcome: ARAT-VR validity

Regarding the content validity, median difficulty of the 13 ARAT-VR items (0[0 to − 1] to 0[0–1]) evaluated by healthcare professionals was rated as equivalent to the classical ARAT for all tasks except those involving the marbles. When manipulating marbles, median virtual task difficulty was rated as superior to the real tasks (1[0–1] when pinching with the thumb-index and thumb-middle fingers, and 1[0–2] when pinching with thumb-ring finger). More specifically, for all items, upper limb sub-movements involved in the ARAT-VR such as hand opening, releasing and object displacement were rated as equally difficult as those involved in the ARAT. However, holding of virtual objects (1[0–1]) and the dexterity needed for pinching movements (1[1–1.5]) were rated as more difficult in VR than in the classical ARAT. Lastly, professionals rated the ergonomics of the application with a median score of 4[4–5] out of 5 and the clarity of the instructions with a median score of 5[4–5] out of 5. All the scores supporting the data are presented in Additional file 4.

When individuals with stroke performed the test with their paretic hand, the ordinal logistic regression model showed that ARAT-VR score variance was exclusively explained by the variance of the ARAT score (p = 0.004) (Table 3). The correlation was not influenced by other factors of upper limb motor control, age, sex, weight, height, type of stroke, side of stroke, time since stroke onset, the presence of cognitive impairments or the hand affected by the stroke (dominant vs. non-dominant). This allowed analyses with the Spearman correlation between ARAT-VR and ARAT-19 scores.

**Table 3:** Correlation between the variation of the ARAT-VR score and the variation other independent variables

	Estimate	Standard error	p-value
Age (years)	0.017	0.048	0.721
Weight (kgs)	− 0.01	0.032	0.758
Height (cm)	0.134	0.082	0.101
Time since stroke onset (months)	− 0.1	0.012	0.431
Classical ARAT (/57)	0.32	0.11	0.004*
FMA-UL (%)	0.07	0.043	0.105
Sex (M/F)	2.183	1.308	0.095
Side of the stroke (L/R)	1.145	1.394	0.412
Type of stroke (ischemic/hemorrhagic)	1.123	1.297	0.387
Presence of cognitive impairments (yes/no)	1.331	1.385	0.337
Laterality of the affected hand (dominant/non-dominant)	1.057	1.465	0.471

*kg* kilograms, *M* male, *F* female, *L* left, *R* right

*Significant p-value

**Fig. 2:**
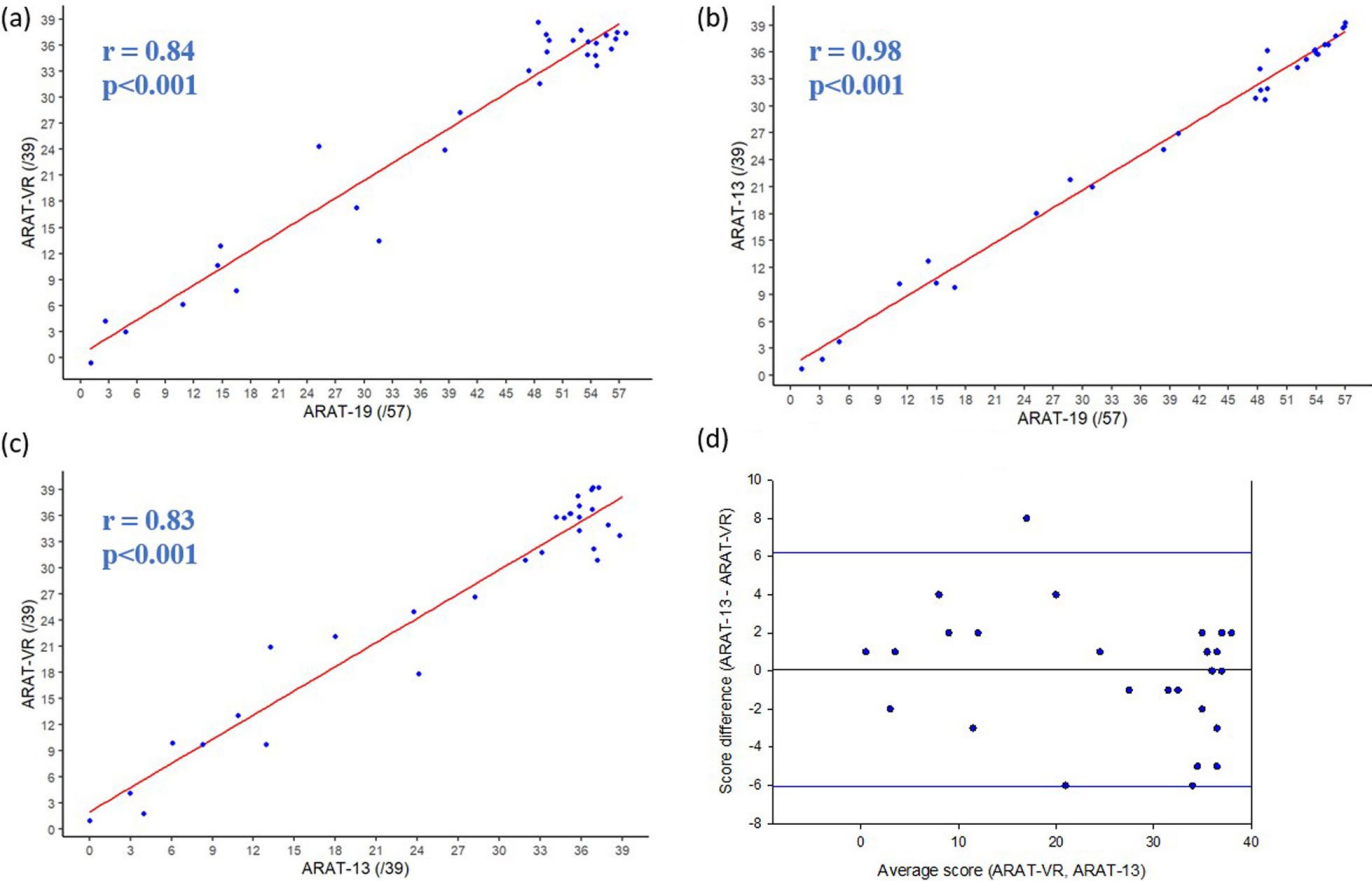
Correlations between ARAT and ARAT-VR scores. **a** In this correlation plot, each point represents paretic hand’s score obtained when performing the ARAT-VR in relation to the ARAT-19 score. Spearman correlation coefficients (r) and their p-value (p) are presented at the left side of the graph. A linear regression is plotted in red. **b** In this correlation plot, each point represents the ARAT-13 paretic hand’s score in relation to the ARAT-19 score. Spearman correlation coefficients (r) and their p-value (p) are presented at the left side of the graph. A linear regression is plotted in red. **c** In this correlation plot, each point represents paretic hand’s score obtained when performing the ARAT-VR in relation to the ARAT-13 score. Spearman correlation coefficients (r) and their p-value (p) are presented at the left side of the graph. A linear regression is plotted in red. **d** In this Bland–Altman plot, each point represents the paretic hand’s average ARAT score, computed by pooling both ARAT-VR and ARAT-13 results, in relation to the paretic hand’s ARAT score difference, computed by subtracting the ARAT-VR to the ARAT-13 results. The horizontal black line represents the mean ARAT score difference and the horizontal red lines, the limits of agreement regarding this mean ARAT score difference (mean difference ± 1.96 standard deviation)

Regarding the concurrent validity, individuals with stroke obtained an ARAT-VR score of 34.5[13–37] out of 39, an ARAT-19 score of 48.5[23–54] out of 57, and an ARAT-13 score of 32[16.75–36] out of 39 for responses made with the paretic hand. There were strong correlations between the ARAT-VR and ARAT-19 scores (r = 0.84; p < 0.001; Fig. 2a), and between the ARAT-VR and ARAT-13 scores (r = 0.83; p < 0.001; Fig. 2c). In addition, the scores of the ARAT-VR and ARAT-13 (out of 39) were similar (Wilcoxon p = 0.765; Bland–Altman mean difference = 0.07 [95% limits of agreement: − 6.044; + 6.178]; Fig. 2d). The ARAT-19 scores were also strongly correlated with the ARAT-13 scores (r = 0.98; p < 0.001; Fig. 2b). Lastly, when taking each item individually, all virtual and traditional item scores were significantly correlated for responses made with the paretic hand (0.45 ≤ r ≤ 0.86; p ≤ 0.01) (Additional file 5). When using the less-affected hand, individuals with stroke obtained an ARAT-VR score of 36[34–37], an ARAT-19 score of 57[56.75–57] and an ARAT-13 score of 39[38.75–39].

HCS obtained an ARAT-VR score of 36[35–38] out of 39 when performed with the dominant hand and of 36[34–38.5] when performed with the non-dominant hand, whereas the HCS obtained an ARAT-19 score of 57 and an ARAT-13 score of 39 with both dominant and non-dominant hands. To understand the influence of experience with technologies on VR performance, we then compared the age of each HCS to their ARAT score difference (when subtracting the ARAT-VR score from the ARAT-19 score). We observed a significant negative moderate correlation between age and ARAT score difference for the non-dominant hand (r = 0.45; p = 0.02) but no significant correlation for the dominant hand.

### Secondary outcomes

Concerning the usability of the ARAT-VR, all participants rated it as excellent on the SUS: 82.5[78.75–87.5] for HCS and 82.5[75–90] for individuals with stroke (Wilcoxon p = 0.946). The lowest score was shown for the 4th item of the scale (entitled ‘I think that I would need the support of a technical person to be able to use the system’). The median score obtained for to this item was of 2[1–3], corresponding to a neutral opinion.

Regarding the inter-session test–retest reliability, results are presented in Table 4. Individuals with acute or subacute stroke were re-seen after a maximum of 3 days and a minimum of 24 h after the first session. Individuals with chronic stroke were re-seen after a maximum of 2 weeks and a minimum of 24 h, and HCS were re-seen approximately 3 months after the first assessment. Results demonstrated excellent reliability between Trials 1 and 2 for paretic hand assessment (ICC = 0.99; p < 0.001), with no significant difference between the scores (Wilcoxon p = 0.945). The MDC was 4.0 for the paretic hand. However, for assessment of the less-affected hand, there was a slight significant improvement between Trials 1 and 2 (median difference = 2.5[1–3]; p = 0.004). For HCS who participated to the retest session, no significant score difference was found between the two trials of the ARAT-VR for both dominant (p = 0.813) and non-dominant hands (p = 0.69).

**Table 4:** Test–retest reliability

	ARAT-VR score Trial 1 (/39)	ARAT-VR score Trial 2 (/39)	ICC	p-value (ICC)	p-value (Wilcoxon)
Paretic hand	32.5[9.25–35.25]	32.5[7.5–35.75]	0.98	< 0.001*	0.945
Less-affected hand	35.5[33.75–36.25]	38[34.75–39]	/	/	0.004*
Dominant hand	36.5[34.25–37.75]	36[34.5–38]	/	/	0.813
Non-dominant hand	36.5[34.5–37.75]	35[33.25–38.5]	/	/	0.69

*ICC* intraclass correlation coefficient

*Significant p-value

Concerning the ARAT-VR execution timing, the median duration was of 3.5[2.49–5.88] minutes for the paretic hand. The duration of the installation of the headset was ~ 1.5 min, the explanation of instructions was ~ 2.5 min, and the learning period was ~ 1 min, making the total administration time of the ARAT-VR to be around 9–10 min.

## Discussion

To the best of our knowledge, this study is the first to provide an immersive virtual version of the ARAT using hand-tracking technology. Furthermore, the number of studies interested in validating other upper limb motor assessments in immersive VR among individuals with stroke remains limited [29]. The present ARAT-VR was found to be valid, usable and reliable to assess the activity of the paretic hand among individuals with stroke.

Due to limitation in the present hand-tracking technology in VR, the number of items of the ARAT had to be reduced in the ARAT-VR (from 19 to 13 items). However, despite this reduction, results confirmed that there was no loss of information. Indeed, as presented in Fig. 2, we observed an excellent correlation (r = 0.98; p < 0.001) between the ARAT-19 (/57) and ARAT-13 scores (/39) for the paretic hand of individuals with stroke. Although Guttman scaling was not used for this experiment, the ARAT-VR application would allow to reduce the time of assessment by automatically managing item selection according to the traditional method [38]. Furthermore, other researchers proposed to reduce the ARAT items from 19 to 4 tasks using a decision tree [39, 40]. It might therefore be interesting to integrate similar decision trees in the ARAT-VR application to further increase time efficiency.

### Difference between virtual and real environment

Individuals with stroke obtained similar scores between the ARAT-VR and the ARAT-13 for the paretic hand. In addition, for all items, several specific ARAT and ARAT-VR upper limb sub-movements (hand opening, releasing and object displacement) were rated as equally difficult. However, most HCS and individuals with stroke using their less affected hand did not achieve the maximal score in VR, whereas all HCS obtained 57/57 on the ARAT-19. This difference between virtual and real environment assessments was further underlined by previous research conducted in immersive VR [28, 29]. Two hypotheses may be put forward to explain these differences. First, it could be that the absence of tactile feedback in immersive VR while manipulating virtual objects may be responsible for this difference. Several studies have pointed out the importance of sensory-tactile input on digital grasping movement performance [41–43]. A lack of tactile feedback can be compensated by other inputs such as visual and proprioceptive feedback, though these compensatory inputs can also show differences. For instance, a virtual Box and Block Test using vibrating feedback showed score differences between real and virtual environments [29]. Second, the fact that most HCS did not reach the maximal score in VR may be explained by their age, with the data showing a negative correlation with score difference between the ARAT-VR and ARAT-19. The affinity for technology tends to decrease with age [44], and in this study, some of the HCS discovered virtual and hand-tracking technology for the first time. Indeed, although the total SUS score was found to be excellent, the 4th item SUS results suggested that some participants would need close support when starting to use the virtual test in the future. In this case, the familiarization period might not have been sufficient to exhaust the learning effect.

### Reliability

This study showed excellent test–retest reliability, with significant correlations and no significant differences between the scores of the first and second trials of the ARAT-VR when performed with the paretic hand among individuals with stroke. The MDC was 4.0 for the paretic hand and was slightly superior to the traditional ARAT (MDC = 3.0) [45]. The reliability and MDC of the ARAT-VR, obtained among a subgroup of participants, should be confirmed on a greater number of individuals. In addition, further trials may be conducted to measure the minimal clinically important difference (MCID) for the ARAT-VR in individuals with stroke.

### Clinical implications

The implementation of the virtual tests in clinical routine may offer several interesting perspectives. First, functional assessments developed in immersive VR could allow individuals to be evaluated remotely and more autonomously, potentially leading to an increased frequency of assessments as encouraged by current guidelines [2]. The ARAT-VR could therefore be seen as an alternative approach to the traditional ARAT, offering the possibility for objective evaluation, made without the need for clinician presence. Motion capture cameras and inertial measurement units of immersive headsets also offer the opportunity to provide objective data such as kinematics characterizing the movement quality. These measures are important in the evaluation of functional recovery, as a score in the ARAT can be obtained from a range of movements with different qualities. In addition, these additional measures may allow to differentiate real upper limb motor function recovery, typically characterized by an increased movement smoothness and linearity, from compensation, often associated with irregular movements and subnormal activity of other body parts such as the trunk [5, 46].

The reduction of items offers interesting perspectives for clinical routine and research. Indeed, there is a growing demand for shorter and more efficient versions of the ARAT. Yet, as presented in the results, the median execution timing of the ARAT-VR was found to be around 4 min. When considering the duration of the installation of the headset (~ 1.5 min), the provisioning of instructions (*~* 2.5 min) and the learning period (~ 1 min), the total administration time of the ARAT-VR could be estimated to be around 9–10 min whereas, in clinical routine, the ARAT-19 requires up to 15 min depending on the scoring methods used [3, 6]. Moreover, in the ARAT-VR, the total number of items could be reduced to 4, by integrating recently validated decisions trees [39, 40].

The ARAT-VR could also serve as a basis to integrate and validate existing prognoses models in the future. Algorithms already exist for the traditional ARAT and Fugl-Meyer [47, 48]. The integration of such models in VR applications would enable to automatically predict motor recovery leading to a better planning and adaptation of rehabilitation and treatments.

Lastly, implementing virtual tests such as the ARAT-VR in a VR rehabilitation module comprising other assessments and serious games could allow individuals to measure their improvements after performing self- or tele-rehabilitation. All these interventions could be done using the same VR device which could contribute to reduce equipment cost. Moreover, virtual assessments scores might also serve as inputs for serious game regulation to automatically adapt difficulty according to individuals’ performance.

### Acceptability, availability, and sustainability

To implement the use of VR in clinical practice, we must first ensure that the system would be accepted by clinicians and individuals with stroke. However, to date, there remains few data in the literature regarding healthcare professional’s opinions on VR. Broadly speaking, clinicians and individuals with stroke seem to appreciate the motivating aspect of VR and recognize its potential to complement traditional rehabilitation [49, 50]. One study conducted with a non-immersive device revealed that clinicians found VR beneficial and challenging but recognized that a learning period might be needed to well understand the functioning of the device [51]. Another study conducted among older adults showed that their attitude became more positive after being exposed to immersive VR when compared to a standard computer exposure [52]. In contrast, some clinicians remain sceptic with VR devices as they feel less challenged and active than during traditional rehabilitation [49].

### Limits and perspectives

The ARAT-VR has several limitations. First, given that the test was developed using hand-tracking technology, providing of tactile feedback was not possible. Although this does not seem to have impacted the concurrent validity among individuals with stroke, it could be hypothesized that providing tactile feedback would help healthy participants to reach the maximal score. For this purpose, it could be interesting to use instrumental gloves allowing the provisioning of haptic feedback. Second, in the future, the time allowed to reach the maximal score for each item in VR (10 s) could be more accurately determined according to norms obtained among healthy subjects. Third, due to hand-tracking limitations, the number of items of the ARAT-VR had to be reduced. Future research incorporating the use of other headsets comprising more accurate hand tracking or new technologies such as smart glasses or a depth motion camera might be of interest to develop a virtual version of all the 19 ARAT items. This could further improve the ARAT-VR validity.

In terms of methodology, different clinimetric properties of the ARAT-VR have not been assessed during this trial (e.g., responsiveness, MCID). It would be worthwhile to test the ARAT-VR among a larger group of individuals, of different ages, with stroke and with other neurological or motor impairment profiles.

## Conclusion

The ARAT-VR is a valid, usable, and reliable tool to assess upper limb activity among individuals with stroke using their paretic hand. This new VR test holds potential to be used, both in clinical and research practice, as an alternative of the traditional ARAT.

## Supplementary Information

**Additional file 1.** STROBE statement checklist.

**Additional file 2.** Movie representing the ARAT-VR.

**Additional file 3.** Content validity questionnaire.

**Additional file 4.** Raw data supporting the results of conclusions of this work.

**Additional file 5.** Correlation results between the scores of each ARAT-VR and ARAT item.

## Data Availability

The dataset supporting the conclusions of this article is included within the article (and its Additional files 1, 2, 3, 4, and 5).

## Abbreviations

ARAT: Action Research Arm Test
VR: Virtual reality
FMA-UL: Upper limb Fugl-Meyer Assessment
ARAT-VR: The immersive virtual reality version of the Action Research Arm Test
ARAT-19: Score of the full Action Research Arm Test computed using a retrospective Gutmann scaling
ARAT-13: Score of the Action Research Arm Test computed by totalizing scores of the 13 items that have been developed in the ARAT-VR only
HCS: Healthy control subjects
SUS: System Usability Scale
ICC: Intra-class correlation coefficients
MDC: Minimal detectable change
