# Supplementary material for: Performing a shortened version of the Action Research Arm Test in immersive virtual reality to assess post-stroke upper limb activity
Source: J Neuroeng Rehabil. 2022 Dec 3;19:133. doi: 10.1186/s12984-022-01114-3 (PMC9719653; doi:10.1186/s12984-022-01114-3)
Supplement: Supplementary file 3 — Additional file 3. Content validity questionnaire. [file 12984_2022_1114_MOESM3_ESM.pdf]

ARAT-VR Questionnaire

Information :

Name : ………………………………………………… Dominant hand : L / R

First Name : …………………………………………… Weight (kg) : ……………

Date of birth : …… / …. / …………… Height (cm) : …………….

E-mail : ……………………………………………….

Hand used : L / R

1. **ARAT-VR vs ARAT**

For each item and for each movement, please compare your impression regarding the difficulty between the ARAT-VR and the traditional ARAT according to the scale described below

-2 : Much easier when performed during the ARAT-VR than traditional ARAT

-1 : Easier when performed during the ARAT-VR than traditional ARAT

0 : No difference

1 : More difficult when performed during the ARAT-VR than traditional ARAT

2 : Much more difficult when performed during the ARAT-VR than traditional ARAT

Grasp :

Cube - 10 cm

Hand's opening movement to grasp the cube

-2	-1	0	1	2

Hand's closing movement to grasp the cube

-2	-1	0	1	2

Object maintaining during the cube displacement

-2	-1	0	1	2

Shoulder flexion while putting the cube on a height

-2	-1	0	1	2

Hand's opening movement to release the cube

-2	-1	0	1	2

- Justification :

....
....
....

Cube - 2.5 cm

Hand's opening movement to grasp the cube

-2	-1	0	1	2

Hand's closing movement to grasp the cube

-2	-1	0	1	2

Object maintaining during the cube displacement

-2	-1	0	1	2

Shoulder flexion while putting the cube on a height

-2	-1	0	1	2

Hand's opening movement to release the cube

-2	-1	0	1	2

- Justification :

....
....
....

Cube - 5 cm

Hand’s opening movement to grasp the cube

-2	-1	0	1	2

Hand’s closing movement to grasp the cube

-2	-1	0	1	2

Object maintaining during the cube displacement

-2	-1	0	1	2

Shoulder flexion while putting the cube on a height

-2	-1	0	1	2

Hand’s opening movement to release the cube

-2	-1	0	1	2

- Justification :

....
....
....

Cube - 7.5 cm

Hand’s opening movement to grasp the cube

-2	-1	0	1	2

Hand’s closing movement to grasp the cube

-2	-1	0	1	2

Object maintaining during the cube displacement

-2	-1	0	1	2

Shoulder flexion while putting the cube on a height

-2	-1	0	1	2

Hand’s opening movement to release the cube

-2	-1	0	1	2

- Justification :

....
....
....

Grip :

Pooring the glass with a glass of water

Hand's opening movement to grasp the glass

-2	-1	0	1	2

Hand's closing movement to grasp the glass

-2	-1	0	1	2

Object maintaining during the glass displacement

-2	-1	0	1	2

Pronation movement needed to poor the empty glass

-2	-1	0	1	2

- Justification :

....
....
....

Tube - 2.25 cm

Hand’s opening movement to grasp the tube

-2	-1	0	1	2

Hand’s closing movement to grasp the tube

-2	-1	0	1	2

Approach/reaching movement (maintaining)

-2	-1	0	1	2

Pulling on the tube

-2	-1	0	1	2

- Justification :

....
....
....

Tube - 1 cm

Hand’s opening movement to grasp the tube

-2	-1	0	1	2

Hand’s closing movement to grasp the tube

-2	-1	0	1	2

Approach/reaching movement (maintaining)

-2	-1	0	1	2

Pulling on the tube

-2	-1	0	1	2

- Justification :

………
………
………

<u>Ring</u>

Hand's opening movement to grasp the ring

-2	-1	0	1	2

Hand's closing movement to grasp the ring

-2	-1	0	1	2

Approach/reaching movement (maintaining)

-2	-1	0	1	2

Pulling on the ring

-2	-1	0	1	2

- Justification :

....
....
....

Pinch :

Thumb-ring finger

Pinching movement

-2	-1	0	1	2

Object maintaining during the displacement of the marble

-2	-1	0	1	2

Shoulder flexion while putting the marble on a height

-2	-1	0	1	2

- Justification :

………
………
………

Thumb-index

Pinching movement

-2	-1	0	1	2

Object maintaining during the displacement of the marble

-2	-1	0	1	2

Shoulder flexion while putting the marble on a height

-2	-1	0	1	2

- Justification :

……
……
……

Thumb-middle finger

Pinching movement

-2	-1	0	1	2

Object maintaining during the displacement of the marble

-2	-1	0	1	2

Shoulder flexion while putting the marble on a height

-2	-1	0	1	2

- Justification :

....
....
....

Gross movement :

Touching the top of the head

-2	-1	0	1	2

- Justification :

……
……
……

Touching the mouth

-2	-1	0	1	2

- Justification :

……
……
……

General appreciation

On a scale ranging from 1 to 5, where 1 is poor and 5 perfect, could you evaluate the following features of the ARAT-VR :

Ergonomics of the application

1	2	3	4	5

- Justification :

....
....
....

Clarity of instructions

1	2	3	4	5

- Justification :

....
....
....

Thank you for your help !
