# Supplementary material for: Performing a shortened version of the Action Research Arm Test in immersive virtual reality to assess post-stroke upper limb activity
Source: J Neuroeng Rehabil. 2022 Dec 3;19:133. doi: 10.1186/s12984-022-01114-3 (PMC9719653; doi:10.1186/s12984-022-01114-3)
Supplement: Supplementary file 5 — Additional file 5. Correlation results between the scores of each ARAT-VR and ARAT item. [file 12984_2022_1114_MOESM5_ESM.pdf]

Cube - 10cm

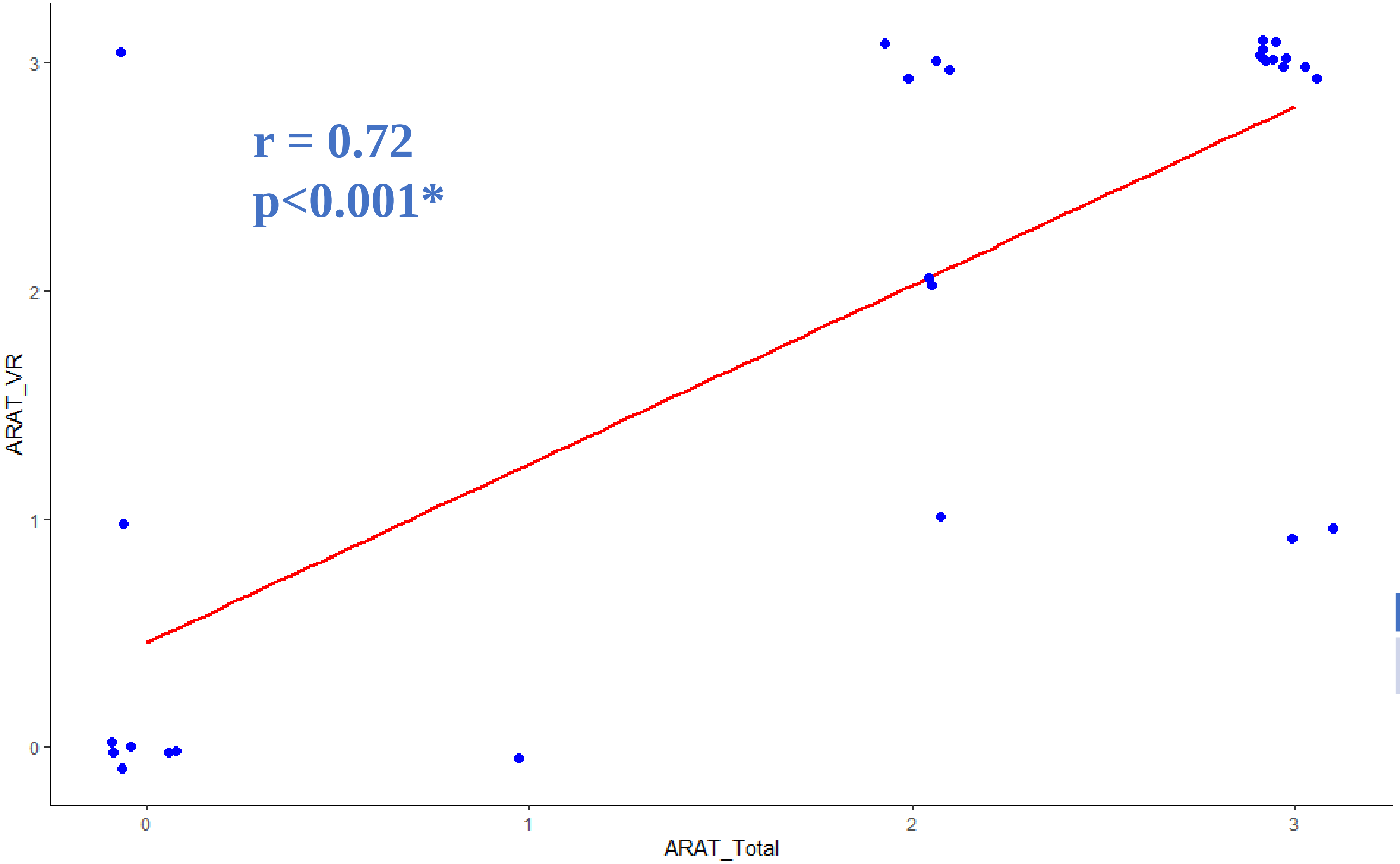

Wilcoxon signed rank test

ARAT_VR	ARAT(/39)	p-value
3 [0 - 3]	2 [0 - 3]	0.846

Cube - 2.5cm

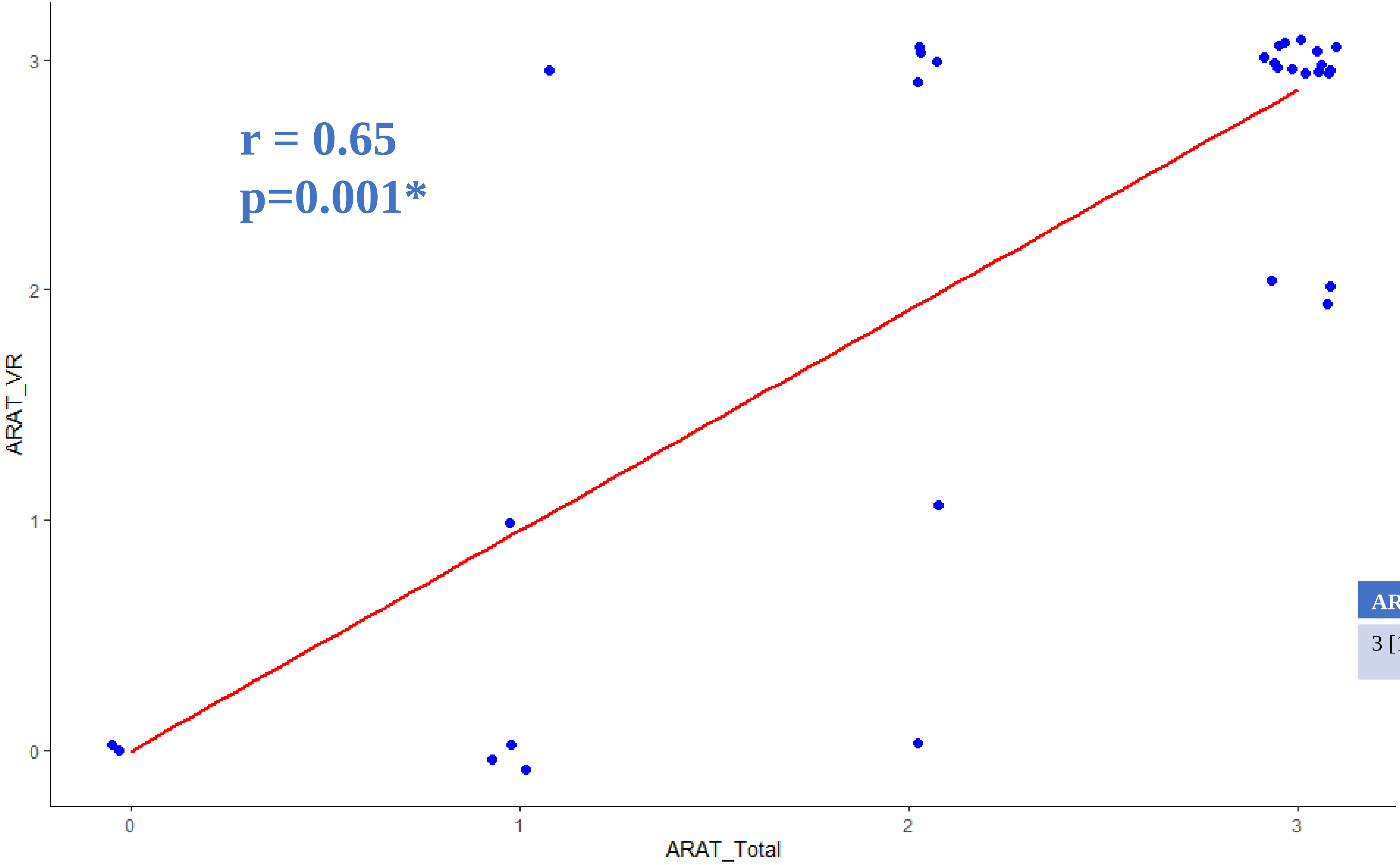

Wilcoxon signed rank test

ARAT_VR	ARAT(/39)	p-value
3 [1 - 3]	3 [1.75 - 3]	0.542

Cube - 5cm

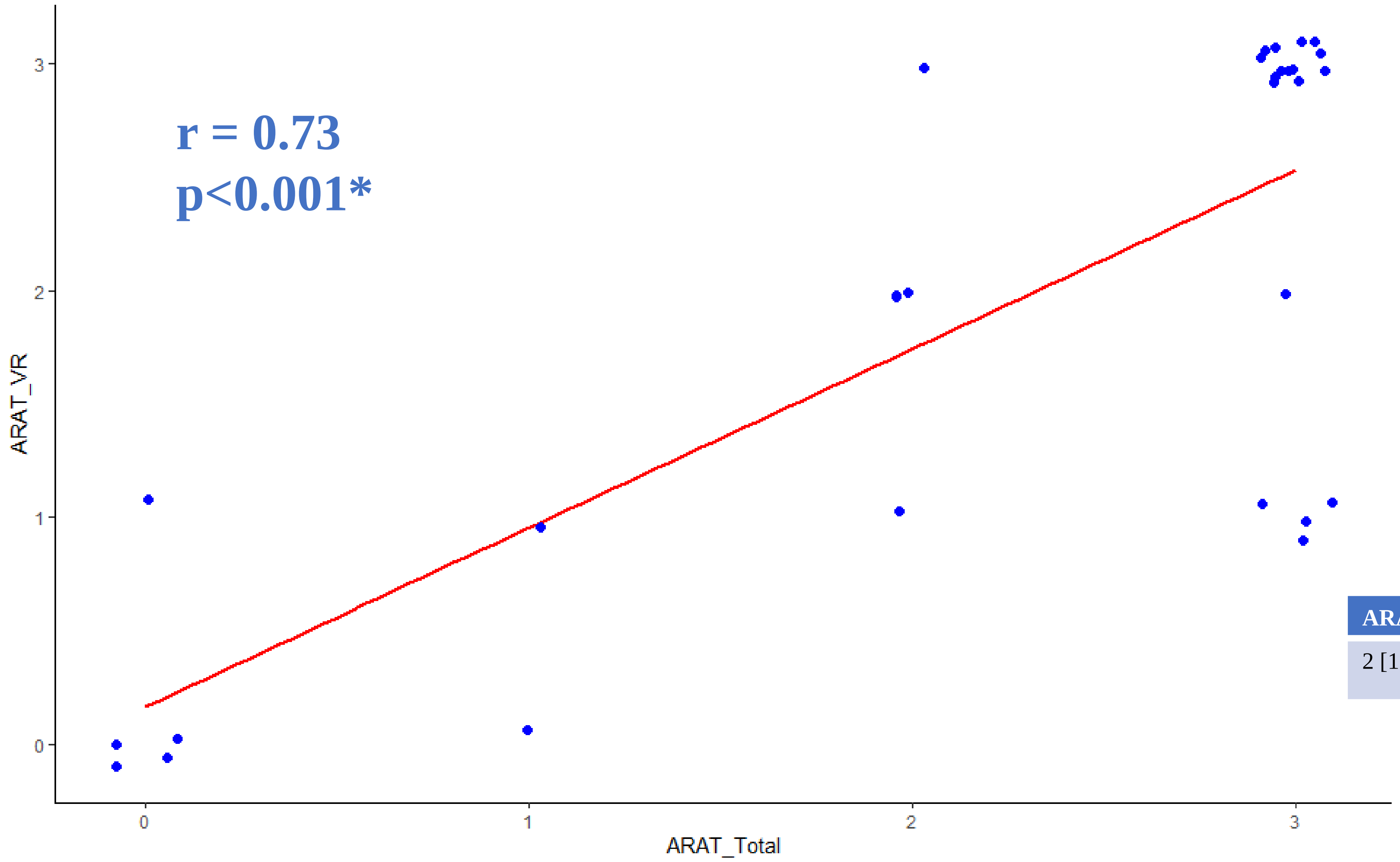

Wilcoxon signed rank test

ARAT_VR	ARAT(/39)	p-value
2 [1 - 3]	3 [1.75 - 3]	0.055

Cube - 7.5cm

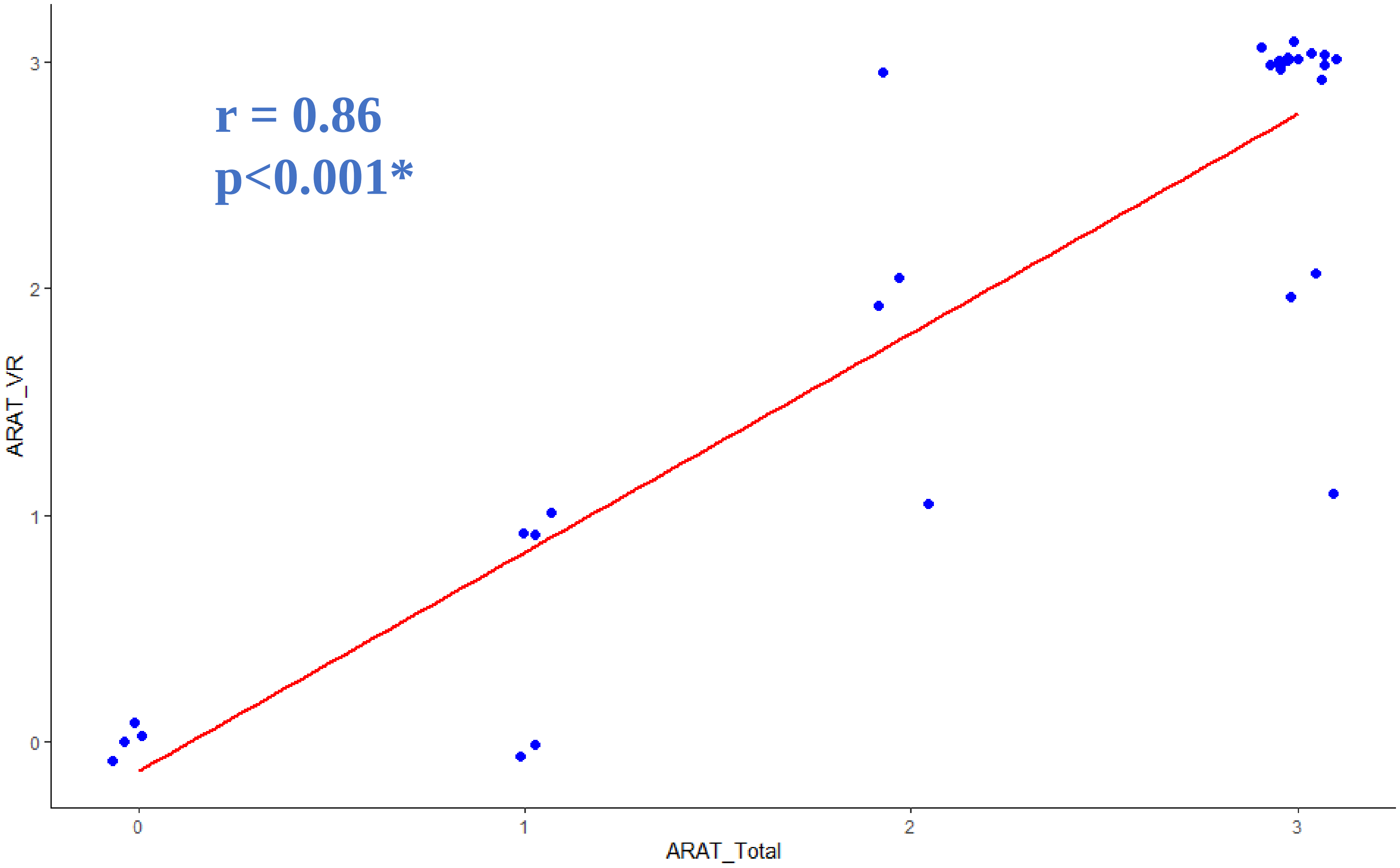

Wilcoxon signed rank test

ARAT_VR	ARAT(/39)	p-value
2.5 [1 - 3]	3 [1 - 3]	0.08

Glass of water

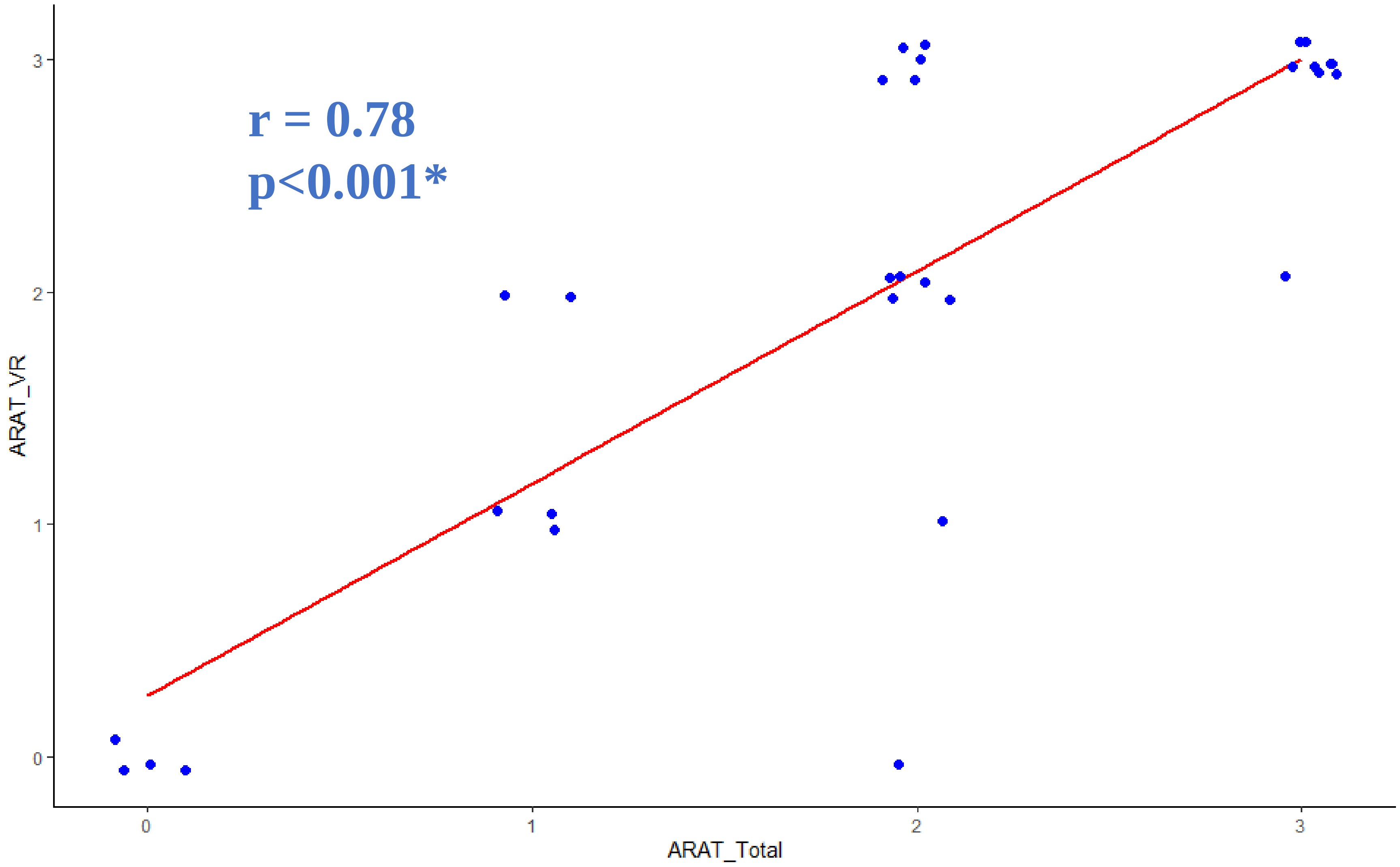

Wilcoxon signed rank test

ARAT_VR	ARAT(/39)	p-value
2 [1 - 3]	2 [1 - 3]	0.492

Tube – 2.25 cm

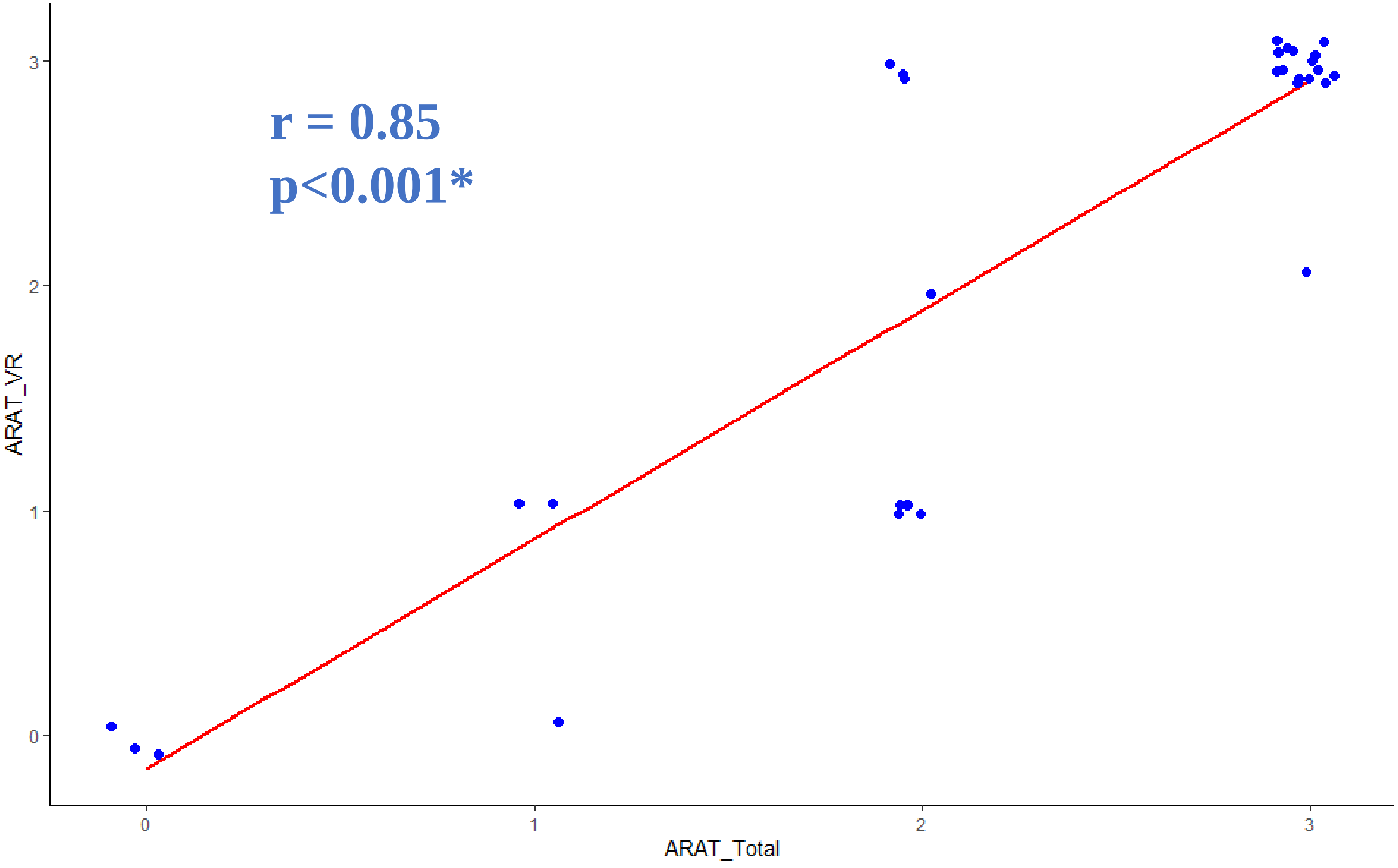

Wilcoxon signed rank test

ARAT_VR	ARAT(/39)	p-value
3 [1 - 3]	3 [2 - 3]	0.426

Tube – 1 cm

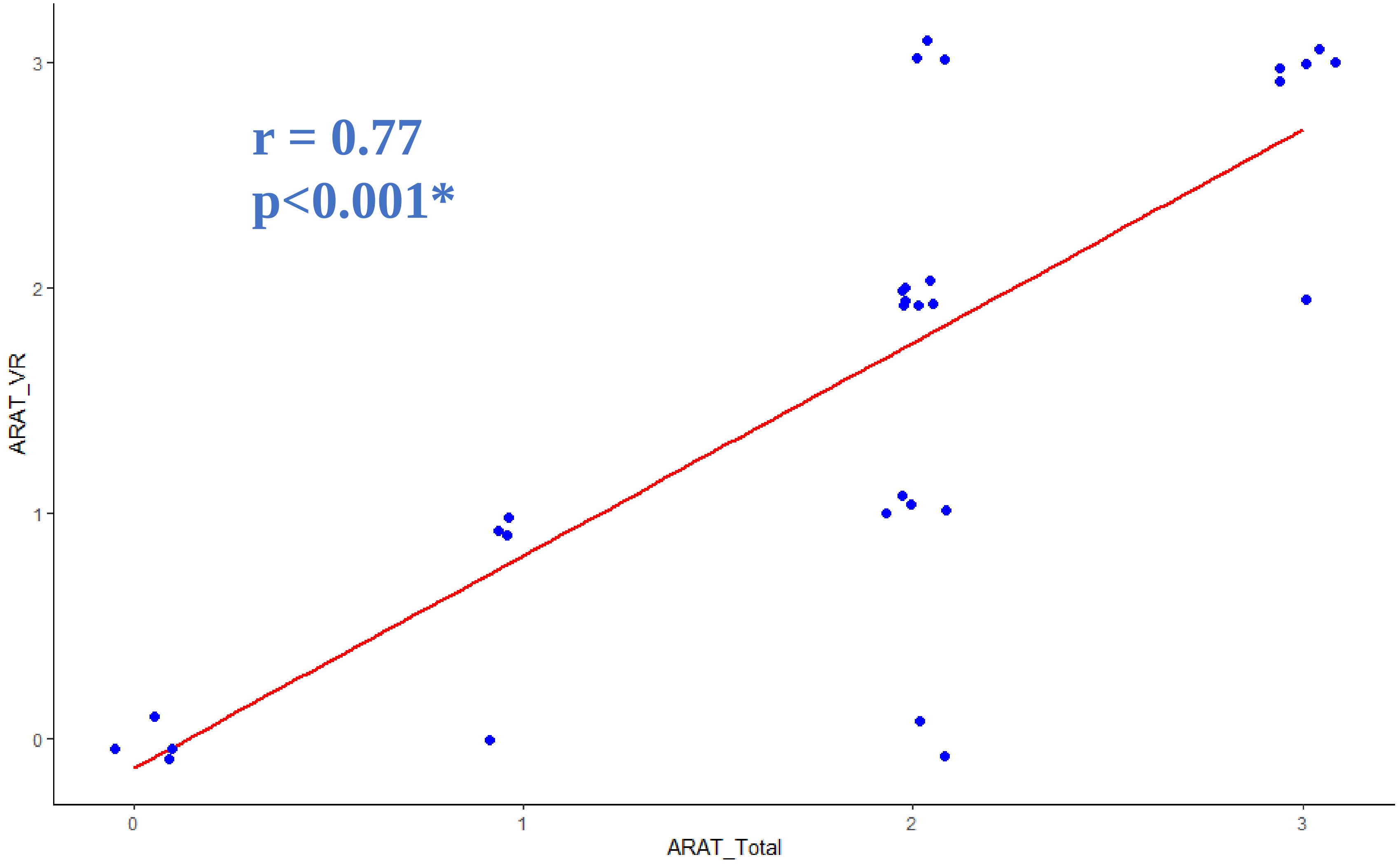

Wilcoxon signed rank test

ARAT_VR	ARAT(/39)	p-value
2 [0.75 - 3]	2 [1 - 2]	0.123

Ring

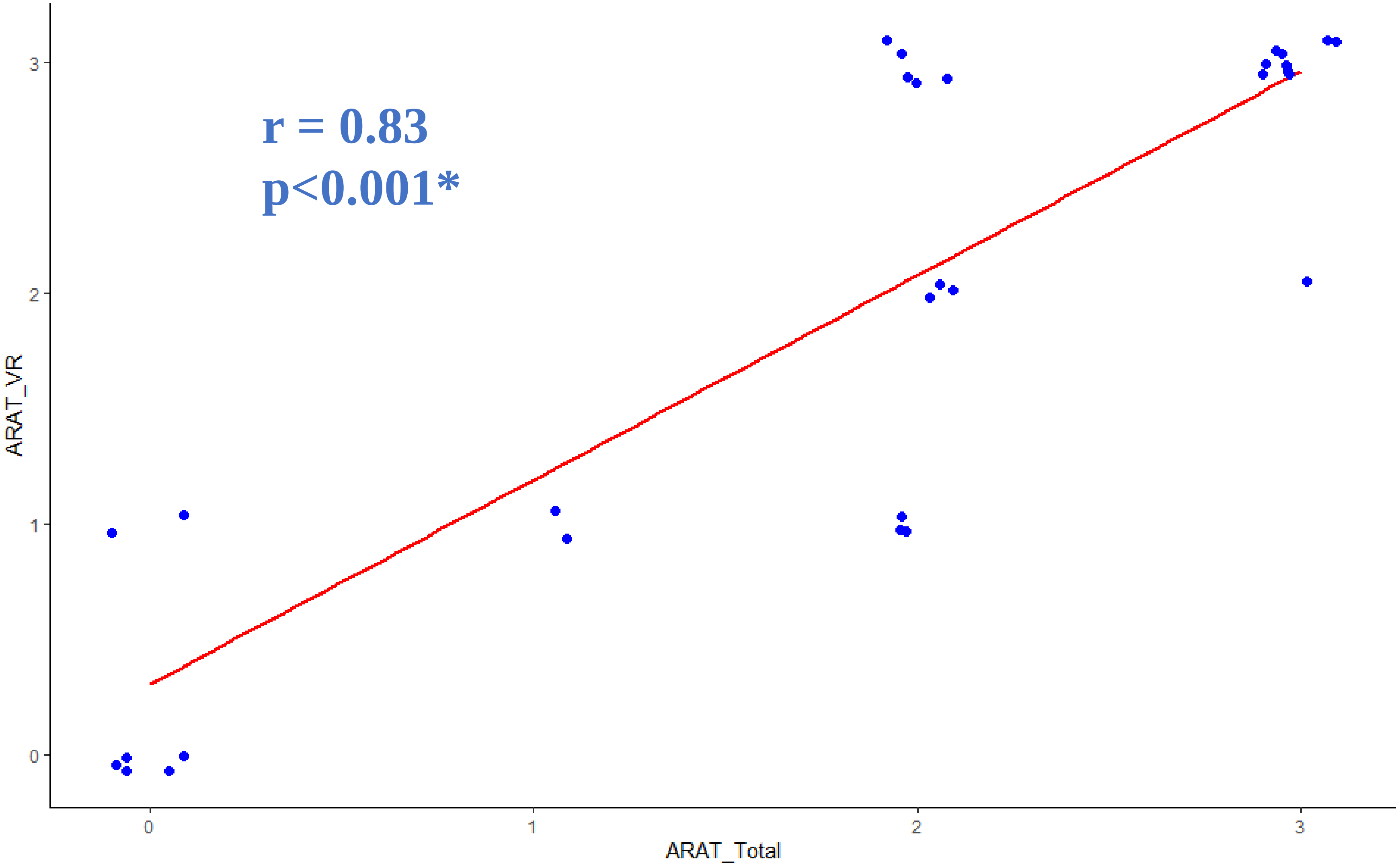

Wilcoxon signed rank test

ARAT_VR	ARAT(/39)	p-value
2 [1 - 3]	2 [0.75 - 3]	0.465

Marble – thumb ring finger

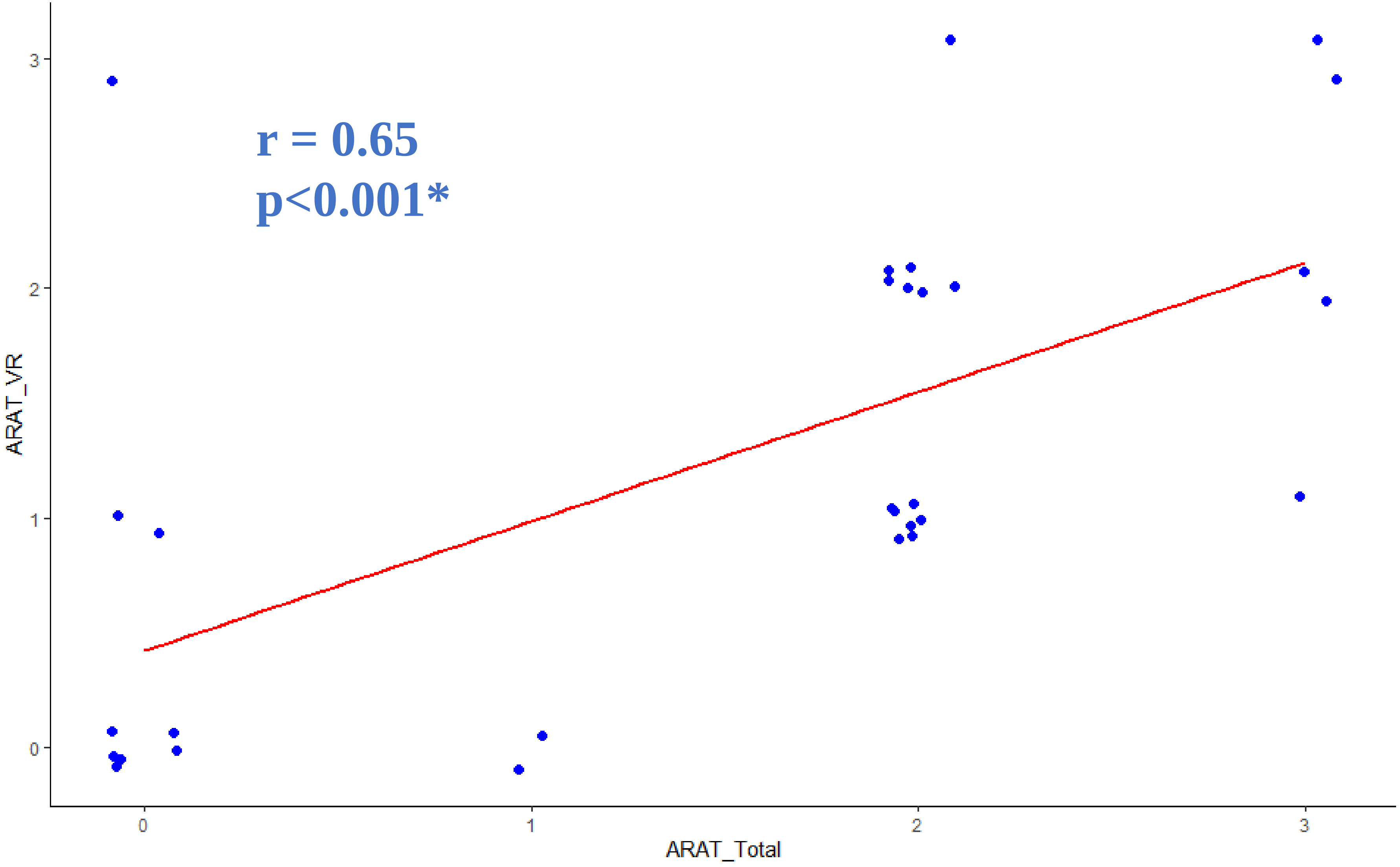

Wilcoxon signed rank test

ARAT_VR	ARAT(/39)	p-value
1 [0 - 2]	2 [0 - 2]	0.130

Marble – thumb-index

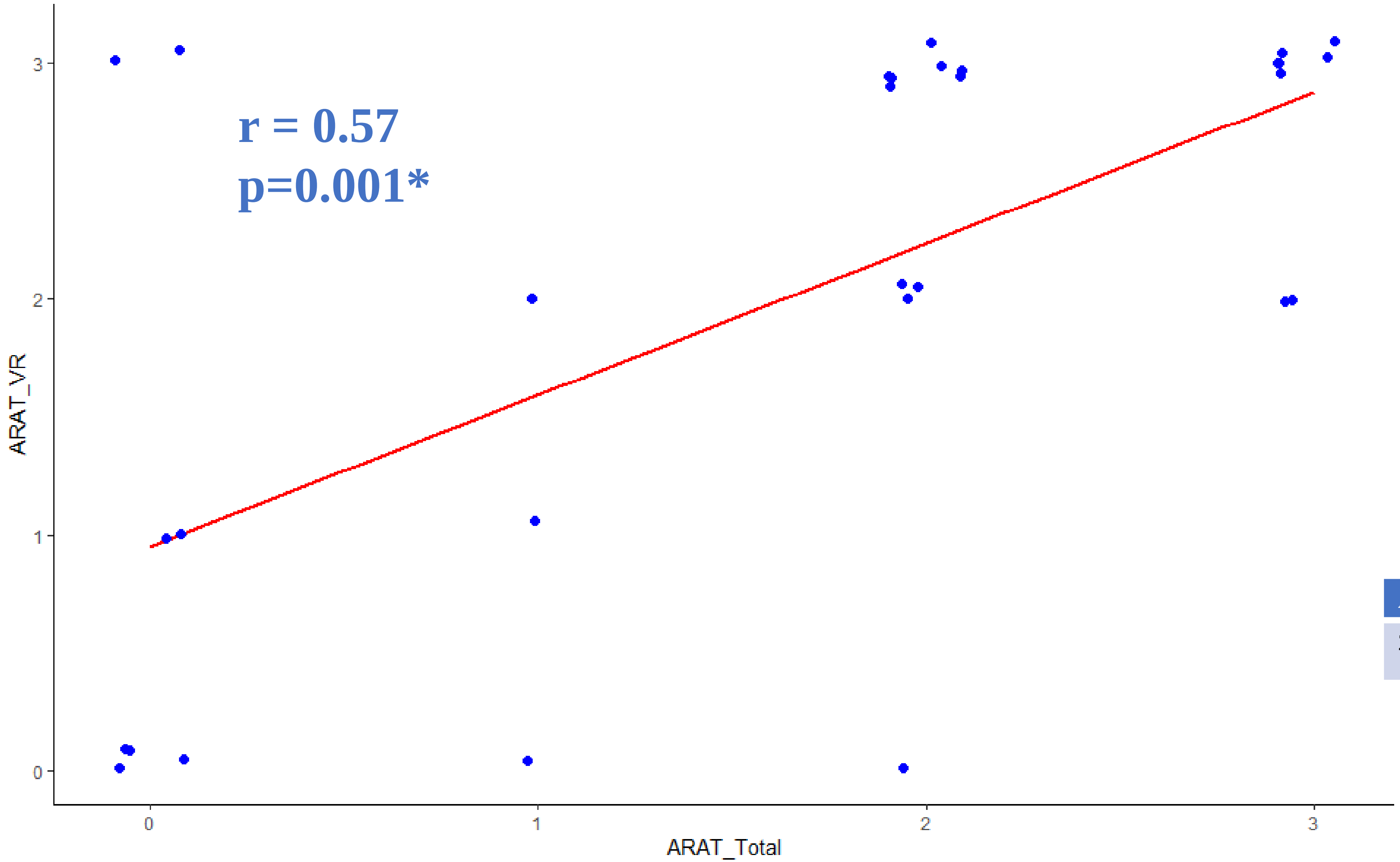

Wilcoxon signed rank test

ARAT_VR	ARAT(/39)	p-value
2.5 [1 - 3]	2 [0 - 3]	0.093

Marble – thumb-middle finger

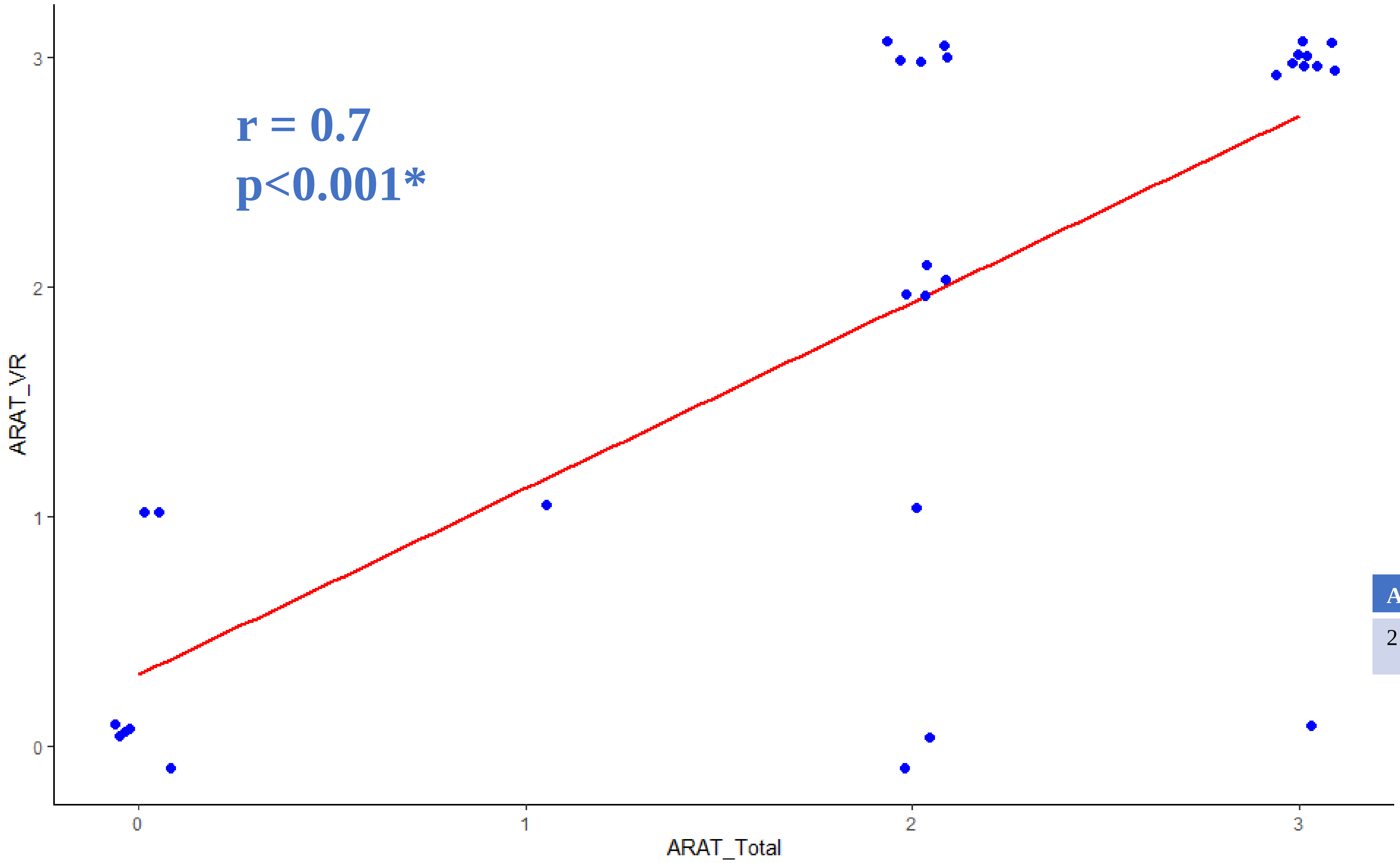

Wilcoxon signed rank test

ARAT_VR	ARAT(/39)	p-value
2 [0 - 3]	2 [0.75 - 3]	0.898

Touching the head with the hand

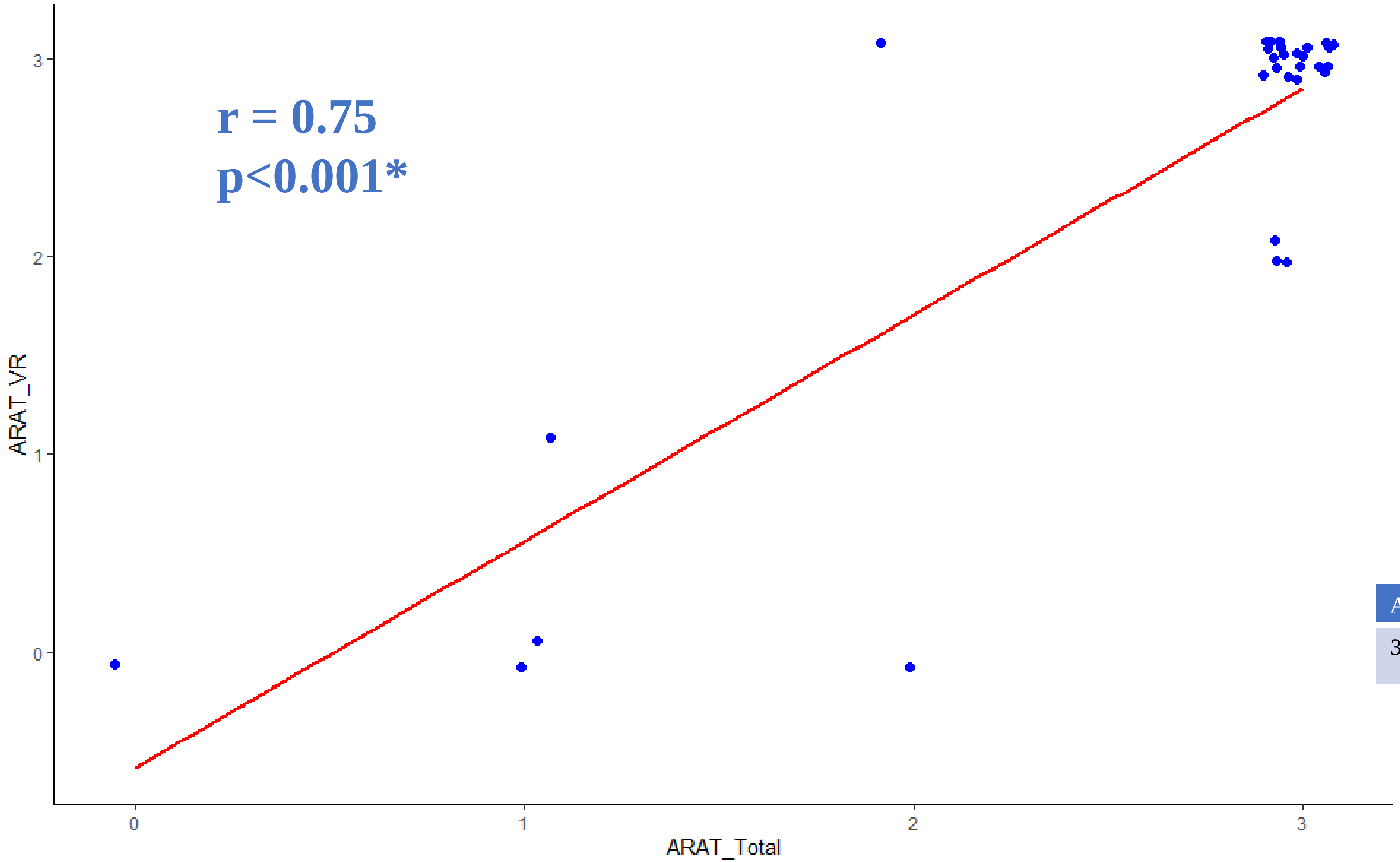

Wilcoxon signed rank test

ARAT_VR	ARAT(/39)	p-value
3 [2 - 3]	3 [3 - 3]	0.08

Touching the mouth with the hand

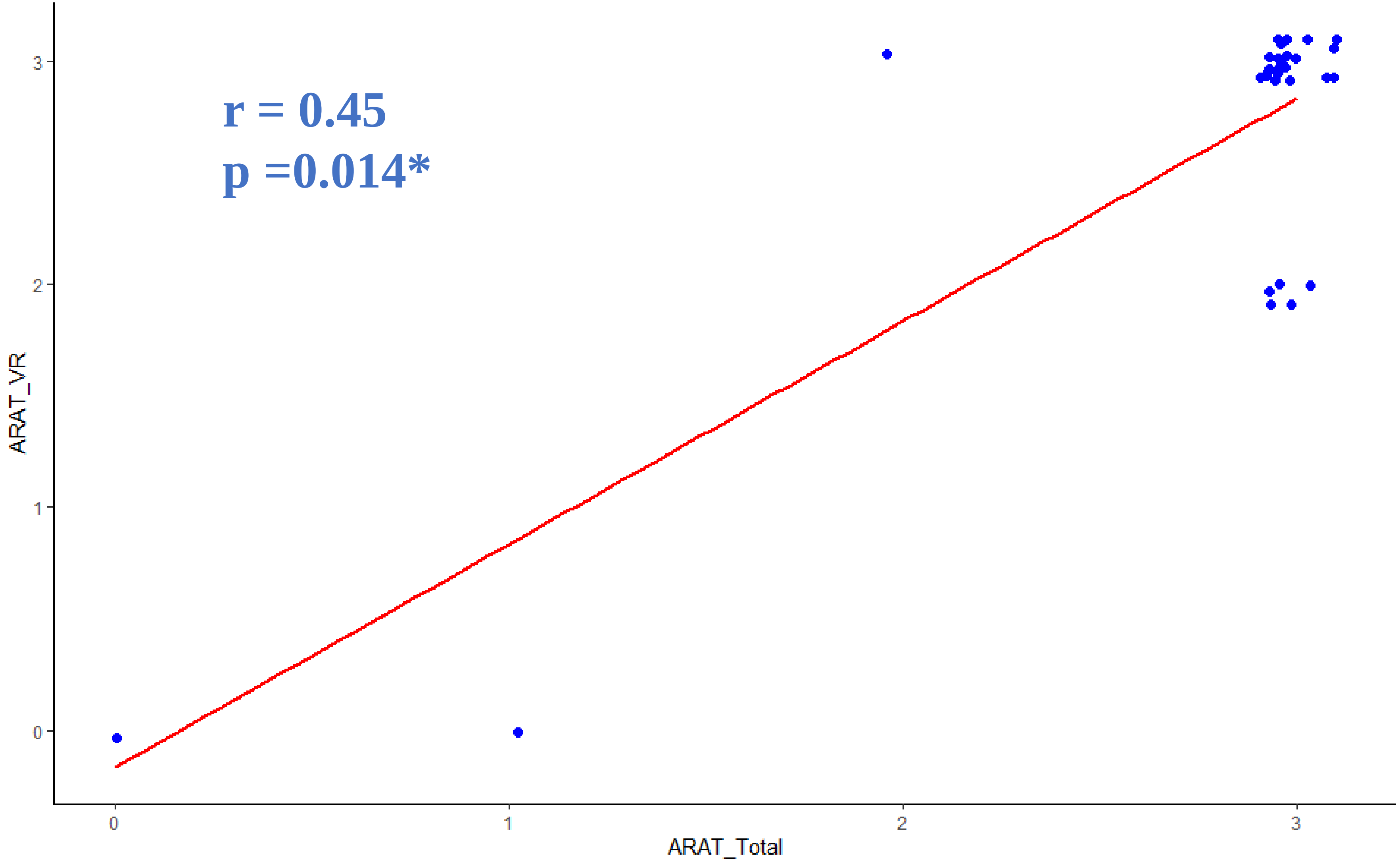

Wilcoxon signed rank test

ARAT_VR	ARAT(/39)	p-value
3 [2.75- 3]	3 [3 - 3]	0.109
